# Early life inter-kingdom interactions shape the immunological environment of the airways

**DOI:** 10.1186/s40168-021-01201-y

**Published:** 2022-02-21

**Authors:** Céline Pattaroni, Matthew Macowan, Roxanne Chatzis, Carmel Daunt, Adnan Custovic, Michael D. Shields, Ultan F. Power, Jonathan Grigg, Graham Roberts, Peter Ghazal, Jürgen Schwarze, Mindy Gore, Steve Turner, Andrew Bush, Sejal Saglani, Clare M. Lloyd, Benjamin J. Marsland

**Affiliations:** 1 Department of Immunology and Pathology, Monash University, Melbourne, Australia; 2 Imperial Centre for Paediatrics and Child Health, Imperial College London, London, UK; 3 Wellcome-Wolfson Institute for Experimental Medicine, School of Medicine, Dentistry and Biomedical Sciences, Queen’s University Belfast, Belfast, UK; 4 Centre for Child Health, Blizard Institute, Queen Mary University of London, London, UK; 5 Human Development in Health School, University of Southampton Faculty of Medicine, Southampton, UK; 6 NIHR Southampton Biomedical Research Centre, University Hospital Southampton NHS Foundation Trust, Southampton, UK; 7 David Hide Asthma and Allergy Research Centre, St Mary’s Hospital, Newport, Isle of Wight UK; 8 School of Medicine, Systems Immunity Research Institute, Cardiff University, Cardiff, UK; 9 Centre for Inflammation Research, Child Life and Health, The University of Edinburgh, Edinburgh, UK; 10 Child Health, University of Aberdeen, Aberdeen, UK; 11 NHS Grampian, Aberdeen, UK; 12 Royal Brompton Hospital, London, UK; 13 National Heart & Lung Institute, Imperial College London, London, UK

## Abstract

**Background:**

There is increasing evidence that the airway microbiome plays a key role in the establishment of respiratory health by interacting with the developing immune system early in life. While it has become clear that bacteria are involved in this process, there is a knowledge gap concerning the role of fungi. Moreover, the inter-kingdom interactions that influence immune development remain unknown. In this prospective exploratory human study, we aimed to determine early post-natal microbial and immunological features of the upper airways in 121 healthy newborns.

**Results:**

We found that the oropharynx and nasal cavity represent distinct ecological niches for bacteria and fungi. Breastfeeding correlated with changes in microbiota composition of oropharyngeal samples with the greatest impact upon the relative abundance of *Streptococcus* species and *Candida*. Host transcriptome profiling revealed that genes with the highest expression variation were immunological in nature. Multi-omics factor analysis of host and microbial data revealed unique co-variation patterns.

**Conclusion:**

These data provide evidence of a diverse multi-kingdom microbiota linked with local immunological characteristics in the first week of life that could represent distinct trajectories for future respiratory health.

**Trial registration:**

NHS Health Research Authority, IRAS ID 199053. Registered 5 Oct 2016. https://www.hra.nhs.uk/planning-and-improving-research/application-summaries/research-summaries/breathing-together/

**Video abstract**

**Supplementary Information:**

The online version contains supplementary material available at 10.1186/s40168-021-01201-y.

## Background

Vast numbers of microorganisms, collectively referred to as the microbiota, reside on our body barrier surfaces, including the skin, gut and airways. The bacterial component of the microbiota has received the most attention due to its high abundance, and in particular, the accessibility of 16S rRNA gene amplicon sequencing and analysis pipelines. However, a wider range of microbes (fungi, viruses, archaea) representing different kingdoms of life also reside in the respiratory tract of healthy individuals, yet little is known about their function, particularly in health. Because they share the same host microenvironments, it is enticing to speculate that these microorganisms interact, compete or even cooperate with each other with potential consequences for the host. It is now well established that the respiratory (bacterial) microbiota develops rapidly after birth, within the first few weeks of life, shaped by the tissue habitat. This pattern has been observed in multiple studies focusing upon the bacterial microbiota of the upper respiratory tract [1–5] and in a single study for the lower airways [6], due to limitations surrounding the invasive sampling procedures in healthy children. While it is clear that niche-specific physiological differences shape the composition of the bacterial microbiota across the length of the respiratory tract in the first weeks of life, whether a similar process operates for other types of microbial life remains to be elucidated.

Subtle changes in the bacterial composition of the upper airways microbiota, or disrupted colonisation patterns in the first year of life, have been linked to susceptibility to respiratory infections [7, 8] and inflammatory diseases later in life, such as wheeze and asthma [2, 7, 9–11]. Yet, the mechanisms underlying the correlations between bacterial taxonomic composition and disease are poorly understood. In addition to providing resistance to invading pathogens and modulating immune responsiveness in inflammatory conditions, the airway microbiota also plays a central role in the development of the immune system during a critical time window in early life. This idea has been supported by experimental studies with germ-free mice, or treatment with broad spectrum antibiotics in early life, which led to enhanced susceptibility to allergic airway inflammation in mouse models of allergic asthma [12–14]. By contrast, mechanistic insights derived from human neonatal data are scarce and are often limited to correlations with broad clinical phenotypes [1, 2, 4, 7, 15].

In this study, we aimed to investigate early-life bacterial and fungal communities in conjunction with the local host immune landscape in the upper respiratory tract of healthy newborns. In essence, we aimed to define the microbial and immunological features of the healthy upper airways. Amplicon sequences of the bacterial 16S rDNA region and the fungal Internal Transcribed Spacer (ITS) region as well as paired host nasal transcriptomics samples were analysed from 121 1-week old neonates of the Breathing Together Study cohort [16]. Here, we provide evidence of a diverse multi-kingdom microbiota in the upper airways of healthy newborns linked with discrete immunological profiles.

## Results

### Study design and quality control

**Fig. 1:**
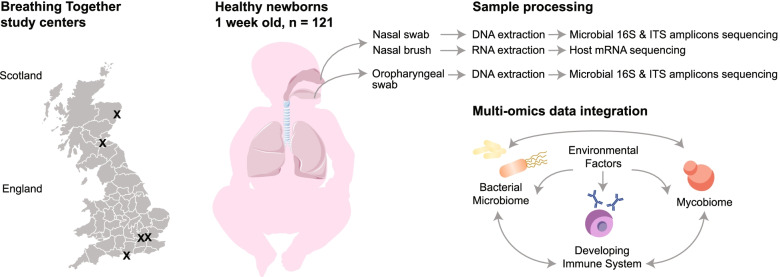
Study design. 121 healthy 1-week-old newborns were prospectively enrolled from different recruiting centres in Scotland (Aberdeen, Edinburgh) and England (Imperial College London, Queen Mary University London and Isle of Wight). Participants were sampled across two respiratory sites, the nostrils and oropharynx, for both bacterial and fungal targeted amplicon sequencing. An additional set of samples was acquired from the nostrils for host gene expression analyses. Resulting datasets and relevant metadata were integrated to address the impact of cross-kingdom associations on the developing respiratory immune system

We enrolled healthy newborns of the ongoing prospective Breathing Together birth cohort, aiming to investigate the pulmonary epithelial barrier and immunological functions at birth and in early life [16]. We applied very strict postnatal age selection criteria to ensure samples were as comparable as possible; specifically, samples from 121 participants taken within a tight time window of 6 to 9 postnatal days were included in the study. Detailed characteristics of study participants are presented in Supplementary Table 1. Neonates were sampled across 2 upper respiratory niches, the nasal, and oropharyngeal cavities, for multi-kingdom microbiota profiling (Fig. 1). Additional samples were simultaneously obtained from the nasal cavity, predominantly consisting of epithelial cells, for host gene expression analyses.

**Fig. 2:**
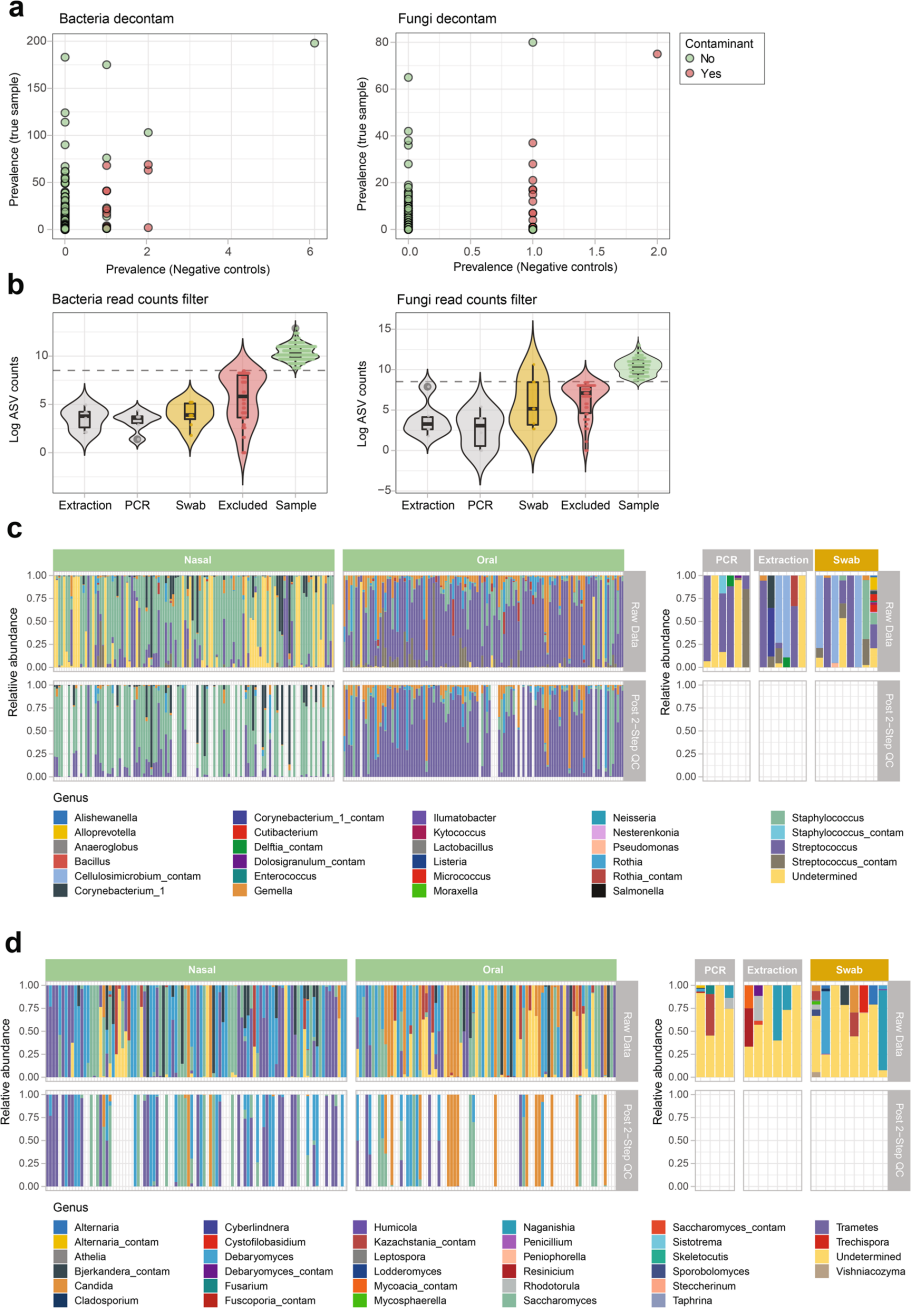
Quality control and decontamination of microbiota samples. **a** Scatter plots showing the prevalence of bacterial and fungal taxa in samples versus negative controls (extraction and PCR water controls), with taxa in red representing those that were identified as contaminants and those in green representing taxa retained for downstream analyses. **b** Violin plots with log-transformed bacterial and fungal read counts for extraction, PCR water and swab controls, a summary of read counts for excluded samples in red, and samples in green. **c** Relative abundance data for bacterial taxa in each sample both in the raw data and following the 2-step quality control measures (removal of contaminants and filtering by read counts, abundance and prevalence). Nasal and oral samples are shown on the left, and controls are shown on the right. **d** Corresponding relative abundance data for fungal taxa

Controlling for contaminants in low biomass samples, such as neonatal airways swabs, is key to prevent biases due to possible bacterial DNA contamination from extraction and other processing reagents. This also applies to DNA of fungal origin, which has received far less attention. To minimize the risk of contamination, all laboratory processing steps were carried in a DNAse and UV-treated laminar flow hood. In addition, 3 types of negative controls *n* = 6 PCR controls, *n* = 6 DNA extraction controls, *n* = 8 “air” swabs (Eswab opened and closed in the room where the sampling took place) were processed alongside clinical specimens at different processing stages, including sequencing. Both samples and taxa were quality filtered using a 2-step approach. First, potential contaminant Amplicon Sequence Variants (ASVs) were identified and removed using the decontam R package [17]. 28 bacterial and 16 fungal ASVs were identified as contaminants (Fig. 2a) and subsequently removed from the sequencing datasets. In a second step, samples falling below a minimum ASV count threshold of 5000 were excluded from the dataset Fig. 2b. None of the negative control samples (PCR, DNA extraction) passed the read count filter step, giving confidence that the ASVs passing the 2-step filter represent valid microbial signals from the upper respiratory tract. Similarly, none of the swab negative controls (air) passed the quality control filter with the exception of one for fungal amplicon sequences. 66% (78/118) and 57% (68/119) of the nasal respiratory niche samples passed the quality filtering for bacteria (Fig. 2c) and fungi (Fig. 2d) amplicon data, respectively. Differences between bacterial and fungal data filtering were more pronounced in the oropharyngeal respiratory niche samples with 88% (105/119) of samples passing the filter for bacterial data and only 37% (44/119) for fungi. Bacterial microbiota data consisted of 536 ASVs after filtering distributed over 8 bacterial phyla with Firmicutes, Actinobacteria and Proteobacteria being the most abundant phyla and *Streptococcus*, *Staphylococcus*, *Gemella*, *Rothia*, and *Corynebacterium* the most prevalent genera (Fig. 2c). Comparatively, fungal microbiome data consisted of 397 ASVs representing two fungal phyla, Ascomycota and Basidiomycota, with genera *Candida*, *Debaromyces*, *Trametes*, *Wickerhamomyces*, and *Rhodotorula* being the most prevalent (Fig. 2d).

### Bacterial and fungal community constituents are shaped by the local respiratory habitat

**Fig. 3:**
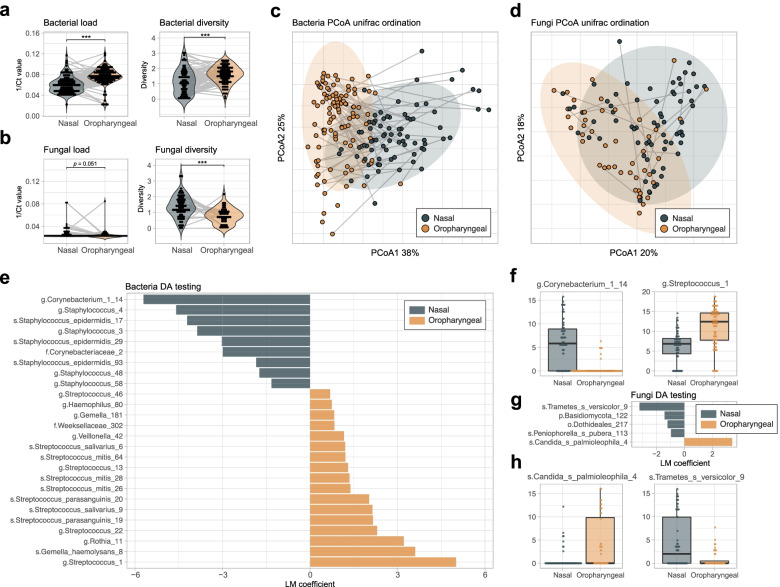
Bacterial and fungal community structure in the nasal and oropharyngeal respiratory niches. **a** Violin plots representing bacterial load measured by quantitative PCR and bacterial diversity (Shannon index) for samples of the nasal and oropharyngeal habitats. **b** Corresponding violin plots for fungal amplicon data. **c** PCoA on the weighted UniFrac distances shown along the first two principal coordinates for bacterial amplicon data. Ellipses represent the 95% confidence interval around the group centroid. **d** Corresponding PCoA for fungi amplicon data. **e** Bacterial signature amplicons comparing the nasal and oropharyngeal niches using MaAsLin for Differential Abundance testing (DA) adjusted for sampling and processing variation. Only significant taxa with a *p* value < 0.05 are shown. **f** Normalised relative abundance of the top 2 bacterial signature taxa of the nasal and oropharyngeal niches. Boxplots represent the median and interquartile range with whiskers determined by Tukey’s method. **g** Fungal signature amplicons comparing the nasal and oropharyngeal niches using MaAsLin. **h** Normalised relative abundance of the top 2 fungal signature taxa of the nasal and oropharyngeal niches. Sample sizes for all panels are *n* = 78 for nasal habitat bacterial data, *n* = 105 for oropharyngeal bacterial data, *n* = 68 for nasal habitat fungal data and *n* = 44 for oropharyngeal fungal data. Colors are representative of the nasal (grey) and oropharyngeal (orange) samples with grey lines linking samples obtained from the same individual. Statistics represent the result of non-parametric Wilcoxon Rank Sum testing for panels **a**–**b** with *p* value < 0.05, < 0.01 and < 0.001 represented as *, ** and ***, respectively

We first aimed to examine the impact of upper respiratory tract niches on bacterial and fungal community structure. Both bacterial load (Wilcoxon, *W* = 2843, *P* = 1.517e−15) and bacterial diversity (Wilcoxon, *W* = 2610, *P* = 2.801e−05) were significantly higher in the oropharyngeal habitat when compared to the nasal habitat (Fig. 3a), a finding previously observed in samples from young children [18] and adults [19]. Comparatively, the mycobiome showed the opposite results with a significant decrease in fungal diversity (Wilcoxon, *W* = 2244, *P* = 8.456e−06) in the oropharynx when compared to nasal samples (Fig. 3b). No clear differences were observed for fungal load analysis (Wilcoxon, *W* = 7847, *P* = 0.052), likely due to the majority of samples falling below the detection threshold. Principal Component Analysis (PCoA) on weighted Unifrac distance between samples showed that bacterial composition was driven primarily by the habitat (Fig. 3c), consistent with previous studies [4, 5, 15]. This was also the case for fungi despite a bigger taxonomic overlap between the nasal and oropharyngeal fungal communities (Fig. 3d). Permutational Multivariate Analysis of Variance (PERMANOVA) further demonstrated that samples clustered by respiratory niche, with a larger *R*^*2*^ value for the bacterial kingdom (PERMANOVA, *R*^*2*^ = 4.7%, *adj. P* < 0.001) than for the fungi one (PERMANOVA, *R*^*2*^ = 3.7%, *adj. P* < 0.001) indicative of a stronger microenvironmental pressure for the bacterial community. Multivariate Analysis by Linear Models (MaAsLin) was performed to infer niche-specific taxa. We identified 26 significant niche-specific bacterial taxa (Fig. 3e) with several *Corynebacterium* and *Staphylococcus* genera being characteristic of the nasal niche, and *Streptococcus* of the oropharyngeal habitat. Top bacterial nasal and oropharyngeal specific taxa were undefined species of *Corynebacterium* (MaAsLin, *coef* = -5.712, *P* = 3.366e−23) and *Streptococcus* (MaAsLin, *coef* = 5.006, *P* = 1.101e−11) genera, respectively (Fig. 3f). In comparison, only 5 niche-specific taxa were detected in the fungal community (Fig. 3g). *Candida palmioleophila* (MaAsLin, *coef* = 3.419, *P* = 8.378e−06) and the environmental fungi *Trametes versicolor* (MaAsLin, *coef* = −3.210, *P* = 1.776e−04) were found to be fungal signature taxa of the oropharyngeal and nasal respiratory sites, respectively (Fig. 3h). Of note, relative abundance levels of *Trametes versicolor* ASV, an environmental lignicolous fungal species commonly detected in house dust [20, 21] and air [22, 23], was not due to contamination in the processing steps (Supplementary Fig. 1b, second panel). Our results suggest that respiratory site-specific microbial communities of different kingdoms emerge within the first postnatal week.

### Multi-omics network inference reveals potential cross-kingdom microbial interactions

**Fig. 4:**
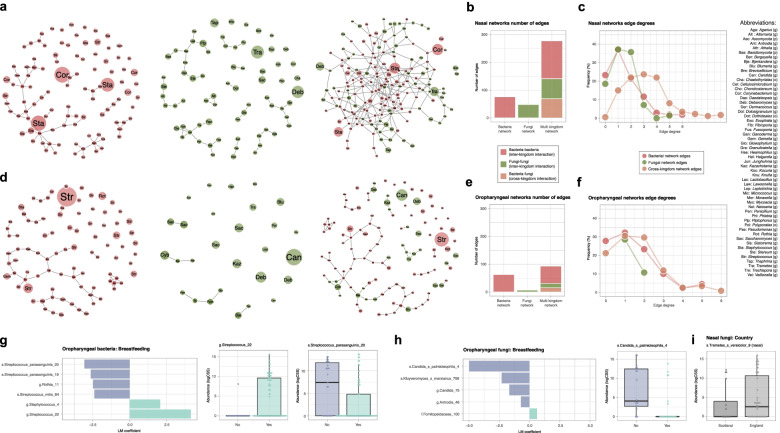
Cross-kingdom microbial interactions in the nasal and oropharyngeal respiratory niches and effect of perinatal factors on nasal and oropharyngeal microbiota composition. **a** Nasal habitat interaction network inferred with SPIEC-EASI for bacteria only (left panel), fungi only (middle panel) and both kingdoms (right panel) on 5% prevalence filtered ASVs. Connecting edges represent significant interactions with node size proportional to ASV average abundance in total samples set and nodes are colored by Kingdom (red color for bacterial ASVs, green color for fungal ASVs) with opacity increasing with closeness centrality. **b** Number of intra- and inter-kingdom edges for each network (bacteria-bacteria in red color, fungi-fungi in green color and bacteria-fungi in salmon color). **c** Frequency of node degrees for each network (red color for bacterial networks, green color for fungal networks, salmon color for multi-kingdom networks). **d**–**f** Corresponding figures for the oropharyngeal cavity. **g** Bacterial taxa associated with breastfeeding or its absence in the oropharyngeal cavity and normalised relative abundance of the top 2 bacterial taxa associated with feeding mode. Boxplots represent the median and interquartile range with whiskers determined by Tukey’s method. **h** Corresponding fungi data. **i** Fungal taxa associated with country factor in the nasal habitat. Sample sizes for the networks are *n* = 51 for nasal and *n* = 39 for oropharynx networks, respectively. Sample sizes are *n* = 78 for nasal habitat bacterial data, *n* = 105 for oropharyngeal habitat bacterial data, *n* = 68 for nasal habitat fungal data and *n* = 44 for oropharyngeal habitat fungal data differential abundance testing

We then aimed to investigate the impact of cross-kingdom interactions on niche-specific community structure using SPIEC-EASI [24] (SParse InversE Covariance estimation for Ecological ASsociation Inference), a sophisticated and compositionally robust statistical framework, which uses sparse graphical model inference. This tool has recently been adapted for multiple kingdoms, allowing the prediction of bacterial and fungal interactions in a study investigating lung and skin microbiota [25]. Inferred nasal networks of bacterial and fungal communities exhibited similar structures; multiple microbial hubs, 10 for bacteria and 12 for fungi (Fig. 4a) with an increased number of edges (connecting lines) for bacteria (*n* = 76 edges) when compared to fungi (*n* = 48 edges) (Fig. 4b). The corresponding multi-kingdom network consisted of a single and densely connected network with 277 edges in total and only 1 disconnected bacterial singleton. 25% were inter-kingdom (bacteria-fungi) connections, indicative of positive co-occurrences between the two kingdoms of life (Fig. 4b). Edge degrees investigation confirmed that the multi-kingdom nasal network was highly connected with an average degree (the number of connections that an ASV has to other ASVs in the network) of 3.2 and a maximum of 9 (Fig. 4c). In comparison, mean edge degrees of single kingdom nasal networks were 1.48 and 1.37 for bacteria and fungi, respectively, with an edge maximum of 5 for bacteria and 6 for fungi. While the oropharyngeal bacterial network was comparable to its nasal counterpart, the oropharyngeal fungal network was different, characterized by no consistent community structure (Fig. 4d). Specifically, known pathogenic taxa with high average relative abundance such as *Candida*, *Debaromyces* or *Saccharomyces* were represented as singletons. Fungal oropharyngeal network average degree was 0.5 with a maximum of 2, a low connectivity also reflected in the cross-kingdom network where only 17% of the edges represented fungi-fungi interactions (Fig. 4e). In the oropharyngeal multi-kingdom network, bacteria-bacteria interactions were the most prevalent (67% of the total number of edges) and overall connectivity was lower than in the nasal equivalent with 93 edges in total, an average edge degree of 1.58 and a maximum of 6. Taken together, these results highlight ecological interactions between bacteria and fungi, particularly within the nasal microenvironment.

### Perinatal factors such as breastfeeding shape both bacterial and fungal microbiota composition

Numerous environmental or birth-related factors have been shown to influence the composition of the upper respiratory tract bacterial microbiota in early-life [1, 2, 4, 15, 26]. We sought to explore how the different respiratory microenvironments responded to selective pressures and whether bacterial and fungal communities responded in a similar manner. Breastfeeding showed the strongest effect on overall bacterial and fungal oropharyngeal community composition (multivariable PERMANOVA, breastfeeding effect *R*^*2*^ = 3.8%, *adj. P* = 0.003 for oropharyngeal bacteria and *R*^*2*^ = 4.7%, *adj. P* = 0.011 for oropharyngeal fungi) (Table 1). Differential abundance testing further corroborated this observation by detecting 6 bacterial and 5 fungal signature taxa related to breastfeeding.

**Table 1:** PERMANOVA and MaAsLin results investigating the effect of perinatal factors on bacterial and fungal microbiota composition for each respiratory site. Multivariable model PERMANOVA results are represented with the effect size (*R*^*2*^) and corresponding *p value*. PERMANOVA results with a *p value* < 0.05 are highlighted with dashed lines. For MaAsLin results, integers represent the number of Differentially Abundant (DA) ASVs for a given factor. Factors of interest with PERMANOVA *p* values < 0.05 and at least 1 differentially abundant ASV are highlighted with dashed lines

	Nasal Bacteria			Nasal Fungi			Oropharyngeal Bacteria			Oropharyngeal Fungi		
	PERMANOVA		MaAsLin	PERMANOVA		MaAsLin	PERMANOVA		MaAsLin	PERMANOVA		MaAsLin
	*R2*	*P-value*	*DA ASVs*	*R2*	*P-value*	*DA ASVs*	*R2*	*P-value*	*DA ASVs*	*R2*	*P-value*	*DA ASVs*
**Gestational age at birth**	0.050	0.917	1	0.060	0.369	2	0.065	0.090	*None*	0.075	0.779	*None*
**Delivery mode**	0.006	0.938	*None*	0.012	0.658	*None*	0.009	0.362	*None*	0.018	0.684	*None*
**Breastfeeding**	0.015	0.280	*None*	0.018	0.197	1	0.038	**0.003**	6	0.047	**0.011**	5
**Country**	0.015	0.306	*None*	0.032	**0.007**	1	0.021	**0.048**	*None*	0.038	**0.044**	*None*
**Gender**	0.007	0.902	*None*	0.012	0.679	*None*	0.007	0.512	*None*	0.026	0.290	*None*

Differentially abundant bacterial taxa included members of the *Streptococcus* genus, a trend observed in a recent study investigating both oropharyngeal and nasopharyngeal samples in the first 6 months of life [15]. Notably, the use of ASVs rather than Operational Taxonomic Units (OTUs) allowed us to discriminate between different *Streptococcus* sequence variants at species level. For example 2 ASVs of *Streptococcus parasanguinis* (MaAsLin, *coef* = −3.07/−2.636, *P* = 3.97e−03/1.888e−02) and 1 of *Streptococcus mitis* (MaAsLin, *coef* = −2.39, *P* = 2.92e−03) were discriminant taxa for non-breastfed neonates while another *Streptococcus* ASV (undefined at species level) was highly abundant in samples from breastfed children (MaAsLin, *coef* = 4.149, *P* = 4.71e−05) (Fig. 4a). Similarly, breastfeeding significantly impacted the composition of the fungal communities in the oropharyngeal cavity (Fig. 4b). Specifically, 2 ASVs belonging to known pathogenic genus *Candida* were most abundant in neonates who were not breastfed. This was particularly notable for *Candida palmioleophila* (MaAsLin, *coef* = −4.99, *P* = 0.002) detectable in 57% of the non-breastfed neonates and completely absent in 88% of those breastfed. Finally, the country (Scotland or England) in which the child was born significantly impacted the overall nasal microbiome fungal composition (multivariable PERMANOVA, country effect *R*^*2*^ = 3.3%, *adj. P* = 0.007) (Fig. 4c). This difference in beta diversity was largely due to differences in the relative abundance of the environmental fungi *Trametes versicolor* (MaAsLin, *coef* = −3.74, *P* = 0.004), previously identified as one of the signature taxa of the nasal cavity. We also confirmed that differentially abundant taxa were either completely absent (most of the cases) or present in significantly lower proportions in negative controls for both bacteria (Supplementary Fig. 1a) and fungi (Supplementary Fig. 1b). In summary, of all the factors tested, breastfeeding had the strongest effect on oropharyngeal microbial colonisation, affecting both bacterial and fungal communities.

### Multi-Omics Factor Analysis reveals potential host-immune interactions

**Fig. 5:**
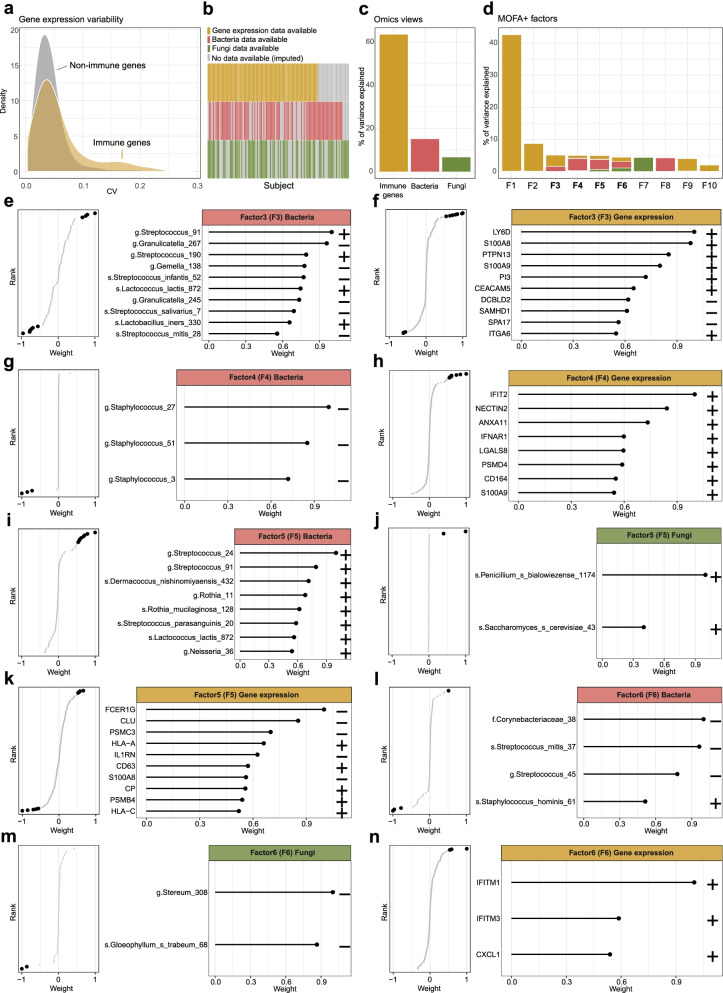
Multi-Omics Factor Analysis (MOFA+) of host immune gene expression and microbiota in the nasal cavity. **a** Gene expression coefficient of variation (CV) of immune (grey) versus non-immune (yellow) protein coding genes. **b** Dataset availability per subject (columns) and omics modality (rows). Unavailable datasets (no sample collected or failed QC) are highlighted in grey. **c** Cumulative proportion of variance explained (*R*^*2*^) by each omics modality. **d** Percentage of variation explained by each factor across the different omics modalities. Factors with more than 2 omics modalities are highlighted in bold. **e**–**n** Loadings of the ASVs and/or genes with the largest weights for a given factor. Yellow color relates to immune genes, red to bacteria and green to fungi. Sample size for MOFA+ analysis is *n* = 109 subjects

We next sought to explore host-microbial interactions within the nasal microenvironment using host transcriptomics. We first examined whether perinatal factors could influence host gene expression independently of microbial colonisation. Unlike the microbial communities, no factor was associated with changes in the expression of protein coding genes. Protein coding genes related to immune responses displayed higher variability in expression when compared to non-immunological genes (Wilcoxon, *W* = 247573, *P* = 8.72e−5), suggestive of varying degrees of immune activation in healthy newborns (Fig. 5a). We hypothesised that the presence of specific microbes in the upper respiratory tract of healthy newborns could shape immune function and explain the high variability observed in expression. Integration of host immune gene expression and multi-kingdom microbiota data was performed using Multi-Omics Factor Analysis (MOFA+), enabling the identification of shared sources of variation and correlation between multi-omics data sets [27, 28]. One advantage of this tool is that the probabilistic framework can handle missing datasets, allowing us to capture information from all available patients (*n* = 109 subjects, *n* = 85 gene expression datasets, *n* = 78 bacterial datasets, *n* = 69 fungal datasets) even if the datasets do not overlap for a given patient due to missing samples or failed QC (Fig. 5b). The cumulative proportion of variance (*R*^*2*^) explained by each omics modality was highest for immune gene expression (63%), followed by bacterial composition (15%) and lowest for fungal composition (7%) (Fig. 5c). While the first 2 inferred factors were principally explained by variation in gene expression, factors 3 to 6 revealed variation that was shared between the omics modalities (Fig. 5d). Loadings of factors explained by at least 2 omics modalities were investigated. Weights analysis of Factor 3 revealed that ASVs of *Streptococcus*, *Lactococcus* and *Lactobacillus* genera (Fig. 5e) were positively associated with lymphocyte antigen 6 family member D (LY6D) gene expression, as well as 2 pro-inflammatory alarmins, S100 Calcium-Binding Protein A8 (S100A8) and A9 (S100A9), known to play a role in the development of neutrophilic asthma [29–31] (Fig. 5f). Genera inversely correlated with factor 3 included two ASVs belonging to *Granulicatella* and two to *Streptococcus* genera, in addition to *Gemella.* Factor 4 was characterized by negative weights for *Staphylococcus* ASVs (Fig. 5g) and positive weights for immune genes related to interferon signalling, interferon-induced protein with tetratricopeptide repeats 2 (IFIT2) and Interferon Alpha And Beta Receptor Subunit 1 (IFNAR1), cell adhesion molecule NECTIN2 and annexin ANXA11 (Fig. 5h). This suggests a negative correlation between specific *Staphylococcus* species, usually considered as “healthy” commensals in the nasal/nasopharyngeal cavities, with proinflammatory interferon signals. Factors 5 captured variation in all 3 omics modalities with top bacterial weights related to *Streptococcus* ASVs, as well as 2 ASVs of *Rothia* genera (Fig. 5i). Of note, 2 of these were previously identified as signature taxa for the oropharyngeal cavity (*Rothia* and *Streptococcus parasanguinis*) (Fig. 3e). Factor 5 positive weights were principally characterised by *Streptococci* and *Rothia* genera with some of the ASVs overlapping with factor 3 (Fig. 5i). The strongest fungal weight associated with factor 5 was a *Penicillium* ASV, followed by *Saccharomyces* (Fig. 5j). Top immune gene weight was linked to Fc Fragment Of IgE Receptor Ig (FCER1G) expression, a receptor for immunoglobulin E (IgE) and a key factor in the pathogenesis of allergic asthma [32]. Other genes with a positive weight associated with Factor 5 included Human Leukocyte Antigen genes A (HLA-A) and C (HLA-C), members of the histocompatibility complex (MHC) class I. Finally, factor 6 was associated with negative weights for bacterial *Corynebacterium* and 2 *Streptococci* (Fig. 5i), as well as environmental fungi *Stereum* and *Gloeophyllum*. With respect to host gene expression, positive weights of 2 Interferon-induced transmembrane protein genes (IFITM1 and IFITM3) were associated with factor 6. Altogether, our data highlighted that variations in the nasal transcriptome of healthy newborns were largely explained by differences in the expression of immunologically relevant genes. Integration of host and microbial data using MOFA+ confirmed that most of the variation between healthy subjects was attributable to differences in immune gene expression followed by bacteria and only moderately to differences in fungal composition. Members of the *Streptococcus* genus were repeatedly linked with variability in immune gene expression, including genes linked with asthma development (alarmins, IgE receptor) or anti-viral immunity (interferon signalling and MHC class I).

## Discussion

While it is becoming increasingly evident that bacteria at mucosal surfaces play a key role in early life immune education, there is a clear knowledge gap on the role of fungi in this process, and a lack of detail concerning the specific immunological pathways that are affected. Mechanistic evidence concerning the role of the gut mycobiome in neonatal immune maturation has just started to emerge [33], and our data now provides insights into the role of fungi in immune development of the airways. Multiple studies have highlighted changes in adult airways mycobiome composition in the context of chronic respiratory diseases such as asthma, cystic fibrosis, bronchiectasis and chronic obstructive pulmonary disease [34, 35]. Understanding the early stages of poly-microbial airways colonisation and its impact on local immunity is key to understanding the contribution of these microbes to respiratory diseases.

We found that both the bacterial and fungal arms of the microbiota develop rapidly after birth (within the first week of life). Similar to what has been described for bacteria [4, 6, 15], the composition of the fungal microbiota is primarily shaped by the respiratory niche. This aligns with the concept that the respiratory tract provides distinct microenvironments along its length harbouring different physiological properties. Specifically, the nasal epithelium represents the first point of contact to the external environment through inhaled air. Large particles are generally stopped by the nasal cilia, while smaller particles such as bacteria or fungi (spores) can be trapped by the surrounding mucus layer. This might explain the presence of airborne environmental fungi such as *Trametes versicolor*, found to be signature taxa of the nasal habitat, which also exhibited significantly higher fungal diversity (and load, to a lesser extent) when compared to the oropharyngeal habitat. This environmental effect was also reflected with differences in beta diversity driven by *Trametes versicolor* when comparing samples from England and Scotland-born babies. Although *Trametes* has been reported in several studies investigating the mycobiome of respiratory samples [36–40], in some cases correlating with health or disease, results should be interpreted cautiously given the potential influence of geographic location. The bacterial component of the microbiota showed the opposite trend; namely a decrease in bacterial diversity and load in the nasal habitat when compared to the oropharyngeal habitat. In fact, the oropharyngeal cavity has been described as the home of the second largest and diverse microbiota community after the gut, given the fact that it provides a rich and stable environment for bacteria in terms of temperature, pH and availability of nutrients [41].

We found that breastfeeding (or its absence) shaped the composition of the bacterial and fungal oropharyngeal microbiota, suggesting that both kingdoms of life are under similar selective pressures. While breastfeeding differences in the bacterial component were mainly driven by distinct *Streptococcus* ASVs, the most striking change in relative abundance was observed for fungi with significant increases in known pathogens related to the *Candida* genera in the absence of breastfeeding. Consistent with these findings, oligosaccharides present in human milk have been shown to reduce *Candida albicans* virulence in epithelial cell cultures [42]. These changes could also be mediated by maternal bioactive molecules such as immunoglobulins (sIgA, IgG, IgM) or other anti-bacterial proteins found in the milk. *Candida* species growth is typically limited by both local immunity and competition with other microbes, such as bacteria. The inability to amplify enough fungal material in 63% of the oropharyngeal samples further reinforces the idea that fungal colonisation may be opportunistic in the presence of a rich bacterial microbiota. This was also reflected in the network inference results, as the highly abundant *Candida palmioleophila* did not cluster with any other fungi in the multi-kingdom network, arguing for competitive or antagonistic interactions.

Multi-kingdom microbial networks inference of the two respiratory tract habitats also revealed potential bacterial and fungal relationships. This was particularly the case in the nasal cavity, probably reflective of a constant influx (inhalation) of microorganisms allowing the cohabitation between the two microbial kingdoms. In contrast, the oropharyngeal network organisation consisted of small disconnected microbial hubs indicative of variable poly-microbial communities, likely to be less resilient to environmental pressures, such as breastfeeding. The nasal mucosa represents the first line of host defense against airborne pathogens, allergens and other foreign particles. In addition to directly secreting antimicrobial components (defensins, lactoferrins, lysozyme, and reactive oxygen or nitrogen species), it plays a role in initiating and controlling immune responses. We found that immune-related genes displayed an increased variability in their expression when compared to non-immune genes. Given the importance of the microbiota in immune maturation, we investigated whether differences in local gene expression were linked with the presence of particular bacterial or fungal microorganisms using multi-omics data integration. Although immune gene expression was the main factor driving heterogeneity between individuals, MOFA+ revealed some factors with shared variation across omics. These mainly involved *Streptococcus* species linked with alarmin signals (S100 genes) and MHC class I genes (HLA genes) or *Staphylococcus* ASVs, whose weights inversely correlated with interferon inducible genes. In comparison, associations between immune gene expression and fungal ASVs were sparse, arguing for a stronger impact of bacteria, as compared to fungi, on host immunity. Two longitudinal studies have linked upper airways colonization with *Streptococcus* in the first months of life with recurrent wheeze and/or asthma at 5 years of age [2, 43]. Given the critical role that type I interferon and MHC I molecules play in antiviral responses and the risk posed by early childhood viral infections for the development of wheeze and asthma [44], the investigation of the respiratory virome represents a key target for future studies.

## Conclusion

In summary, our data reveals the presence of a multi-kingdom microbiota linked with local gene expression in the upper respiratory tract of healthy newborns. We found that the respiratory niche (nostrils versus oropharynx) was the primary determinant of multi-kingdom microbiota composition, as early as 1 week after birth. Breastfeeding impacted the microbiota the most, leading to a shift in both bacterial and fungal microbiota composition, with *Candida* relative abundance dominating in oropharyngeal samples of non-breastfed neonates. Investigation of nasal transcriptome profiles revealed that immune gene expression was highly variable. Multi-omics factor analysis revealed shared sources of variation, particularly linking expression of innate immune genes and bacteria. These findings highlight the importance of considering the microbiota as a dynamic multi-kingdom entity, capable of regulating local immunity, and potentially influencing long term respiratory health.

## Methods

### Participants recruitment and sampling

This study included 121 healthy newborns from the Breathing Together birth cohort [16] sampled 1 week after birth between February 2017 and May 2018. Participants were recruited either antenatally or postnatally in five different centres in the UK (Aberdeen, Edinburgh, Imperial College London, Queen Mary University London and Isle of Wight). Inclusion criteria included term birth (>37 weeks gestational age) and written parental consent. Exclusion criteria for the Breathing Together cohort included multiple pregnancies, positive maternal group B *Streptococcus* from vaginal swab or urine culture, CPAP or ventilatory support, major health problems (e.g. congenital heart disease, cystic fibrosis) and impossibility to follow up within the years of age time frame (e.g. planned relocation). Exclusion criteria for this specific study were antibiotics during pregnancy, newborn in a special care/intensive care unit, complicated vaginal deliveries and any sampling not falling into the 6–9 postnatal days time window. Nasal swabs (taken from both nostrils—swab inserted and rotated 5 times) and oropharyngeal swabs (using a tongue depressor the oropharynx was swabbed by rotating the swab 5 times and avoiding touching the oral cavity) were taken for microbiota analysis using copanusa eSwabs (COPAN Diagnostics) and stored at −80°C. Subsequently, nasal epithelial cells were collected. The infant was held in a supine position and the brush [Interdental brush 2.7 mm diameter (Dentocare 620)] was inserted into the nasal cavity, and directed inferolaterally until resistance was met from the medial aspect of the inferior turbinate. The brush was rotated swiftly three times to obtain cells and removed and placed into an Eppendorf containing 700 μl of RLT lysis buffer (Qiagen) with 2-Mercaptoethanol (Sigma), snap-frozen and stored at −80°C.

### Microbial DNA extraction and sequencing

Samples were centrifuged at 14,000x*g* for 10min at 4°C, and pellets were first incubated with 300U of Lyticase (Sigma) at 37°C for 30min with gentle shaking (500xrpm) to increase fungal DNA recovery. Resulting lysates were further processed using the DNeasy UltraClean Microbial Kit (Qiagen) according to the manufacturer’s protocol, and DNA was eluted in 40μl of microbial DNA-free water (Qiagen). All extraction steps were carried out in microbial DNA-free conditions under a laminar flow hood decontaminated and UV-treated before laboratory processing. To control for potential microbial DNA contaminants, 3 types of negative controls were included: (1) ESwabs negative controls obtained (opening and closing of a tube at the different sampling sites), (2) extraction negative controls (microbial DNA-free water processed through the kit) and (3) PCR negative controls (PCR reaction with microbial DNA-free water instead of DNA template). Positive controls were obtained by processing 5μl of ZymoBIOMICS Microbial Community (Zymo research) similarly to the samples for which we were able to detect 7 out of 8 bacterial species present in the positive control samples. Each sample was amplified in 2 different reactions, the first one with custom barcoded primers targeting the bacterial 16S rDNA v1-v2 region (F-27/R-338) as previously described [45] and the second one with custom barcoded primers targeting fungal Internal Transcribed Spacer region 1 (ITS1) region. Primers were as following:

16S-Forward:

- 5’-AATGATACGGCGACCACCGAGATCTACACTATGGTAATTCCAGMGTTYGATYMTGGCTCAG-3’,

16S-Reverse:

- 5’-CAAGCAGAAGACGGCATACGAGATACGAGACTGATTNNNNNNNNNNNNAAGCTGCCTCCCGTAGGAGT-3’,

ITS-Forward:

- 5’-AATGATACGGCGACCACCGAGATCTACACGGCTTGGTCATTTAGAGGAAGTAA-3’,

ITS-Reverse:

- 5’-CAAGCAGAAGACGGCATACGAGATNNNNNNNNNNNNCGGCTGCGTTCTTCATCGATGC-3’

where the *N* sequences represent the sample-specific 12-nucleotides golean barcodes. Each 25μl PCR reaction consisted of 10.4μl of microbial DNA-free water, 1μl of each primer at 5μM, 2.5μl of Accuprime PCR buffer II (Fisher Scientific), 10μl of DNA template and 1μl of Accuprime Taq polymerase (Fisher Scientific). The cycling parameters were as following: initial denaturation 3min at 94°C, followed by 35 cycles (16S) or 40 cycles (ITS) of 30 s denaturation at 94°C, 30 s annealing at 56°C (16S) or 52°C (ITS) and 60-s elongation at 68°C, with a final extension at 68°C for 10min. Amplicons were quantified using a Fragment Analyzer (Agilent Technologies) with the High Sensitivity NGS Fragment Analysis kit, pooled at equimolar amounts and purified using AMPure XP bead cleanup system (Beckman Coulter). Denatured library pools were sequenced on a MiSeq platform with a MiSeq Reagent Kit v2 (500-cycles).

### Microbial taxonomic profiling

Raw sequences were processed using the microbiome-dada2 pipeline (see code availability section) using the DADA2 (version 1.14.1) [46] R package. Briefly, fastq files were demultiplexed using the iu-demultiplex (version 2.7) function from illumina-utils tools [47], primers and adapters removed with cutadapt [48] (version 2.10), reads filtered and trimmed, sequencing error models generated, sequences dereplicated, amplicon sequence variants (ASVs) inferred, paired-ends merged and chimeras removed. Bacterial 16S ASVs were assigned a taxonomy using the SILVA database train set (version 123) and the SILVA species assignment dataset (version 123) for exact sequence matching. Fungal ITS ASVs were assigned a taxonomy using the UNITE database general release (02.02.2019 version). A phylogenetic tree based on ASV sequences was built by performing a multiple-alignment using DECIPHER R package (version 2.16.1), followed by the construction of a neighbor-joining tree using phangorn R package (version 2.5.5) before fitting a GTR+G+I (Generalized time-reversible with Gamma rate variation) maximum likelihood tree using the neighbor-joining tree as a starting point as previously described [49]. Contaminant taxa were identified using the is Contaminant function of the decontam (version 1.12.0) R package [17]. DNA extraction and 16S/ITS PCR water control samples were used as negative controls, the inverse of the bacterial and fungal load qPCR CT values was used as a measure of DNA concentration, and samples were corrected for batch effects. The decontam method ‘either’ was selected to identify contaminants as those that were either more prevalent in negative controls than true samples (prevalence threshold = 0.5), or features with low frequencies relative to the input DNA concentration (frequency *P* statistic threshold <0.2 or 0.3 for bacteria and fungi, respectively). Contaminant features were then removed prior to filtering. Samples with less than 5000 ASVs were excluded from the dataset and ASVs below 1% prevalence or unassigned at Phylum level were filtered out. Shannon index was determined using the estimate_richness function of the phyloseq (version 1.30.0) [50] R package. ASV counts were normalised using Cumulative Sum Scaling (CSS) using the calcNormFactors function from MetagenomeSeq (version 1.28.2) [51] R package followed by log transformation.

### Microbial load quantification

Total bacterial and fungal loads were determined using a custom multiplex panel with fluorescent probes targeting Pan bacterial 16S and Pan fungal 18S regions. Primers and probes were as follows:

- BactPan-Forward: 5’-TGGAGCATGTGGTTTAATTCGA-3’,
- BactPan-Reverse: 5’-TGCGGGACTTAACCCAACA-3’,
- BactPan-Probe: 5’-CACGAGCTGACGACARCCATGCA-3’ with VIC dye,
- FungiPan-Forward: 5’-GGRAAACTCACCAGGTCCAG-3’,
- FungiPan-Reverse: 5’-GSWCTATCCCCAKCACGA-3’,
- FungiPan-Probe: 5’-TGGTGCATGGCCGTT-3’ with FAM dye.

Each 10μl quantitative PCR reaction consisted of 2.5μl TaqPath 1-Step Multiplex Master Mix (Applied Biosystems), 0.18μl of each primer at 50μM, 0.25μl of each probe at 10μM, 4.28μl of microbial-DNA free water and 2μl of DNA template. Detection was performed using the QuantStudio 6 Flex Real-Time PCR System (Applied Biosystems) with the following conditions: one cycle of 95°C and followed by 45 PCR cycles of 95°C for 15 s and 60°C for 1 min and 15 s. Values are reported as 1/Ct (cycle threshold).

### Host RNA extraction and sequencing

RNA from cell lysates was extracted using the Quick-RNA Microprep kit (Zymo Research) according to the manufacturer’s handbook and eluted in 40μl of RNAse-free water. Library preparation was performed using the QIAseq UPX 3′ Transcriptome Kit (Qiagen) as per the manufacturer’s protocol with 1ng of purified RNA as an input. Briefly, during reverse transcription with template switching, each sample was tagged with a unique molecular index (sample ID) and each RNA molecule with a unique molecular index (UMI). After this step, all the single-tagged cDNA samples were combined into a tube before DNA fragmentation, end-repair, A-addition, adapter ligation and universal library amplification. The denatured library pool was sequenced on a NovaSeq6000 platform with a S1 Reagent Kit (10 -cycles).

### Host transcriptome analysis

Raw 3′ transcriptomics data were processed using the transcriptome-QiaSeq pipeline (see code availability section) using scPipe [52] (version 1.8.0) R package. Briefly, fastq files were reformatted to trim both sample ID and UMI information and include this information in the read header. In the same step, reads with low quality and/or complexity were also removed. Reads were aligned to the GRCh38 human genome using Rsubread [53] (version 2.0.1) R package and counts were assigned based on ENSEMBL (version 98) human gene annotations. The integration of UMI to each transcript allowed a more accurate quantification of mRNA transcripts. All UMI mapping to the same genes and in the same positions were grouped together and duplicated UMIs removed. Ensembl IDs were converted to gene symbols using biomaRt (version 2.45.5) R package. Low abundance genes were filtered using the filterByExp function of EdgeR [54] (version 3.28.1) R package with default settings. Gene counts were normalised using counts per millions (cpm) using the cpm function of EdgeR [54] (version 3.28.1) followed by log transformation. Coefficient of variation (CV) was calculated as following: $CV\;(gene)\;=\frac{(Normalised\;gene\;counts)}{Variance\;(\;Normalised\;gene\;counts)}$. Protein coding genes were retained for downstream analyses. Immune gene list was retrieved from the Immunogenetic-Related Information Source (IRIS) database (December 7, 2014).

### Cross-kingdom interaction analysis

Single-kingdom and cross-kingdom microbial interactions networks were constructed using SParse InversE Covariance Estimation for Ecological Association Inference (SPIEC-EASI) R package [23] (version 1.0.7). Cross-kingdom interactions were inferred using the SPIEC-EASI extension defined by Tipton and colleagues [25]. For microbial networks inference, low abundance ASVs with less than 5% prevalence were filtered out and only samples with matching bacteria and fungi data were used. The Meinshausen-Buhlmann (MB) neighborhood selection method was used, and the optimal sparsity parameter was selected based on the Stability Approach to Regularization Selection (StARS). The StARS variability threshold was set to 0.01 (default) for all networks with 99 repetitions and a nlambda of 20 (default) to achieve network stability. Network layout was defined using the Kamada-Kawai layout algorithm. Inferred networks were imported as graph objects using igraph (version 1.2.5) R package. Edge degree frequencies were calculated using the degree_distribution function in igraph. The parameters were kept the same for all network inferences.

### Multi-omics factor analysis

Multi-omics data integration was performed using MOFA+ tool (version 1.2.2) [27]. Microbial ASVs with at least 5 counts in 5% of the samples were retained for multi-omics data integration and normalised using CLR transformation using microbiome R package (version 1.14.0). Immune gene expression data were normalised using the varianceStabilizingTransformation function of DESeq2 R package (version 1.32.0) [55]. Data and model training options were set to default, and the number of factors was set to 10. All downstream analyses such as the inspection of the top features with largest weights were performed using the plot_weights and plot_top_weights functions of MOFA+ tool (version 1.2.2).

### Statistical analysis

Differences in microbial load and diversity were addressed using non-parametric Wilcoxon Rank Sum testing. Principal Coordinate Analysis (PCoA) was performed on the weighted UniFrac distance of normalised ASV counts using the ordinate function of phyloseq [50] R package (version 1.30.0). Permutational Multivariate Analysis Of Variance (PERMANOVA) was performed on the weighted Unifrac distance of normalised ASV counts with 999 permutations. For the investigation of perinatal factors effects, the model was as follows: ‘Gestational age at birth + Delivery mode + Breastfeeding + Country + Gender’. Differential abundance testing for microbial data was performed by using Microbiome Multivariable Association with Linear Models 2 [56] (MaAsLin2) tool with the following parameters: minimum feature abundance of 1, minimum feature prevalence of 0.1, maximum *q* value threshold value for significance of 0.2 with LM analysis method and Benjamini Hochberg *q* value correction while adjusting for sampling and processing bias. To allow maximal reproducibility in functions requiring random pseudo-numbers, a fixed random seed number was set to 2. Alpha level of significance was set to 0.05 for all statistical tests with *p* value <0.05, <0.01 and <0.001 represented as *, ** and ***, respectively.

## Supplementary Information

**Additional file 1: Supplementary_information.pdf.** Supplementary figures and tables related to the manuscript.

## Data Availability

The data supporting the conclusions of this article are available in the NCBI Sequence Read Archive (SRA) repository, BioProject PRJNA694493 https://www.ncbi.nlm.nih.gov/bioproject/PRJNA694493 including quality controls. Source code for raw microbiota amplicon sequencing data processing can be obtained at: https://github.com/respiratory-immunology-lab/microbiome-dada2. Source code for raw 3’ transcriptome sequencing data processing can be obtained at: https://github.com/respiratory-immunology-lab/transcriptome-QiaSeq.
