# Supplementary material for: Early life inter-kingdom interactions shape the immunological environment of the airways
Source: Microbiome. 2022 Feb 21;10:34. doi: 10.1186/s40168-021-01201-y (PMC8862481; doi:10.1186/s40168-021-01201-y)
Supplement: Supplementary file 2 — Additional file 1: Supplementary_information.pdf. Supplementary figures and tables related to the manuscript. [file 40168_2021_1201_MOESM2_ESM.pdf]

Supplementary Information

Early life inter-kingdom interactions shape the immunological environment of the airways

Metadata	Median (IQ range) / Number (%)
Postnatal age (days)	8 (7-8)
Gestational age at birth (weeks)	39 (39-40)
Weight at birth (gr)	3346 (3042-3672)
Ethnicity	93 White British (77%), Other (23%)
Sex	65 (54%) Female, 56 (46%) Male
Delivery mode	67 (55%) Vaginal, 54 (45%) C-section
Breastfeeding (first week)	87 (72%) Yes, 34 (28%) No
Country of birth	69 (57%) England, 52 (43%) Scotland
Antibiotics during pregnancy	121 (100%) No treatment
Postnatal antibiotics	121 (100%) No treatment

Supplementary Table 1. Participants baseline characteristics. Values represent medians with interquartile range for continuous variables and absolute numbers with percentages for categorical variables.

a

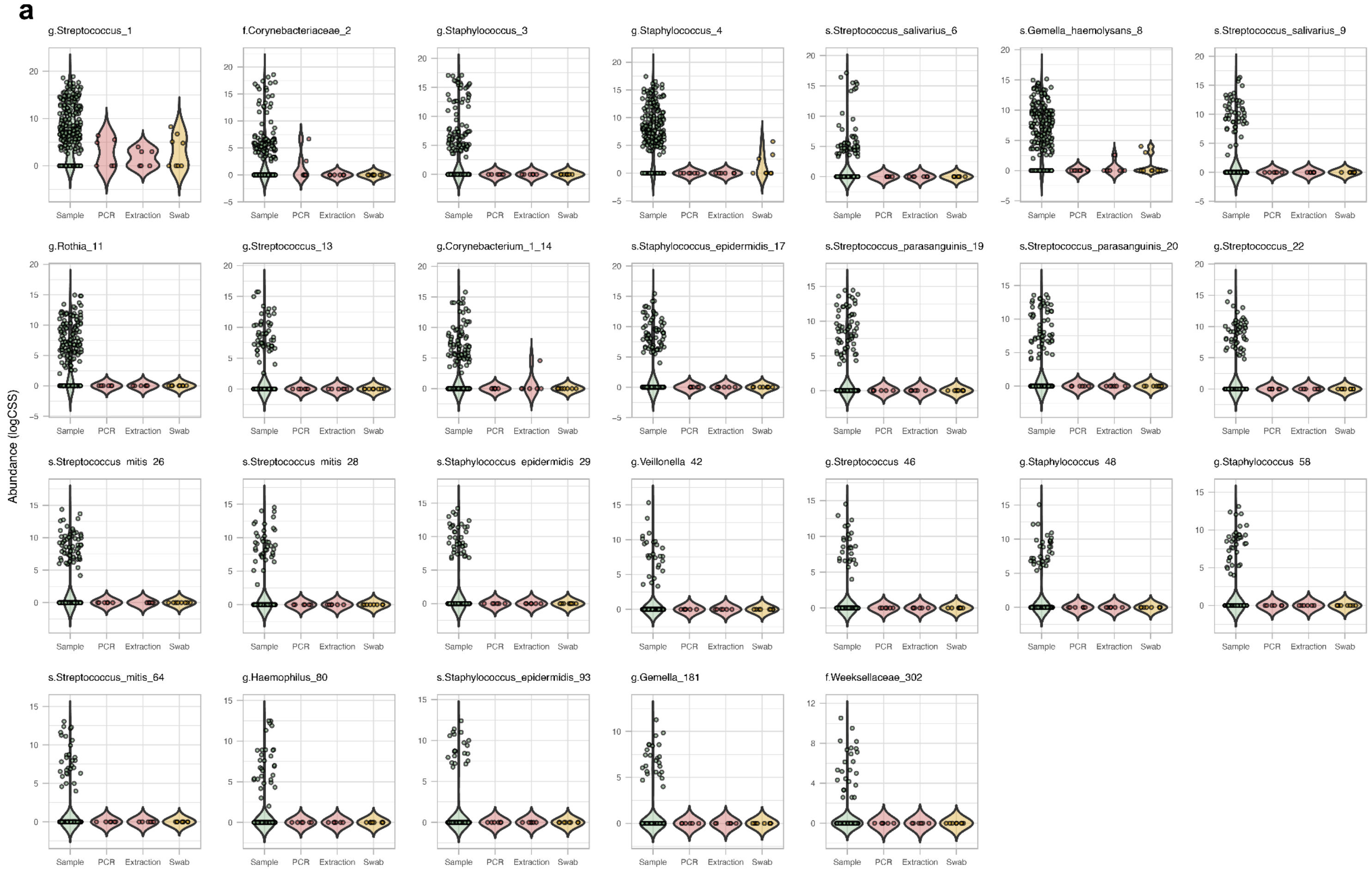

b

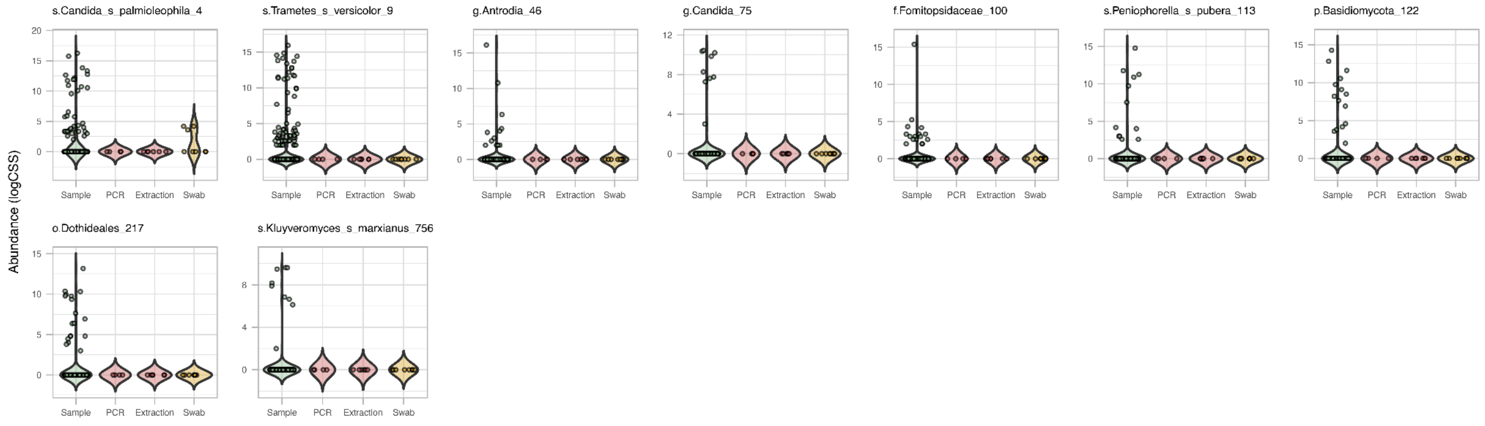

Supplementary Figure 1. a Relative abundance of differentially abundant bacterial genera in samples versus negative controls (logCSS). **b** Corresponding plots for fungi.
